# Gender-based acute outcome in percutaneous coronary intervention of chronic total coronary occlusion

**DOI:** 10.1007/s12471-017-0966-3

**Published:** 2017-02-27

**Authors:** J. E. Guelker, L. Bansemir, R. Ott, K. Kuhr, B. Koektuerk, R. G. Turan, H. G. Klues, A. Bufe

**Affiliations:** 1 Heart Centre Niederrhein, Department of Cardiology, Helios Clinic Krefeld, Krefeld, Germany; 2 Institute for Heart and Circulation Research, University Cologne, Cologne, Germany; 3 Institute of Medical Statistics, Informatics and Epidemiology, University Hospital of Cologne, Cologne, Germany; 4 Department of Cardiology, Krankenhaus Porz am Rhein, Cologne, Germany; 5 University of Witten/Herdecke, Witten, Germany

**Keywords:** Coronary artery disease, Chronic total occlusion, Gender, Acute results

## Abstract

**Background:**

Percutaneous coronary intervention (PCI) of total chronic coronary occlusion (CTO) still remains a major challenge. Insignificant data are reported in the literature about gender differences in CTO-PCI in the era of new drug-eluting stents. In this study we analysed the impact of gender on procedural characteristics, complications and acute results.

**Methods:**

Between 2010–2015 we included 780 consecutive patients. They underwent PCI for at least one CTO. Antegrade and retrograde CTO techniques were applied.

**Results:**

Patients undergoing CTO-PCI were mainly men (84%). Male patients were younger (66.9 years ±10.6 vs. 61.1 years ±10.4; *p* < 0.001), more often smokers, but less frequently had a history of coronary artery disease (24.4% vs. 32.7%; *p* = 0.085) compared with female patients. Female patients more often had diabetes mellitus (29.6% vs. 26.7%; *p* = 0.55) and hypertension (82.7% vs. 80.7%; *p* = 0.55). There were no differences with respect to the amount of contrast fluid, fluoroscopy time and examination time as well as to the length of the stent or the number of the stents. The stent diameter was slightly smaller in women, which was not surprising because the lumen calibre tends to be smaller in women than in men (3.0 mm (2.5–3) vs. 3.0 mm (3–3.5); *p* < 0.001). The success rates were 81.0% in women and 80.1% in men. There was no significant interaction between gender and procedural success and complication rates.

**Conclusions:**

Our retrospective study suggests that women and men have a comparable success rate at a low complication rate after recanalisation of CTO.

## Introduction

Recanalisation of total chronic coronary occlusion (CTO) still remains a major challenge in interventional cardiology. CTO is defined as a complete coronary artery occlusion lasting longer than three months with thrombolysis in myocardial infarction (TIMI) flow grade 0 [1]. The prevalence of CTO has been reported to be up to 30% among patients with a clinical indication for coronary angiography [2]. Due to new interventional techniques and the use of further advanced sophisticated materials, success rates of CTO recanalisation have increased steadily in recent years. In experienced hands reopening rates are more than 85% [3]. Percutaneous coronary intervention (PCI) of CTO is a beneficial tool in coronary artery intervention; if significant myocardial ischaemia is present and there are clinical symptoms due to ischaemia, recanalisation is clearly indicated. The left ventricular function can be improved, more invasive therapies such as coronary artery bypass graft (CABG) surgery can be avoided at lower complication rates and even the prognosis of the disease can be improved in suitable cases with a short-term and long-term survival benefit [4, 5].

Only insignificant data are reported in the literature about gender differences in CTO-PCI. The impact of gender on outcome following CTO-PCI is not well defined. Only outdated data have been published so far. It was reported that the incidence of major adverse cardiac and cerebrovascular events (MACCE) was increased in women compared with men [6]. However, the data were collected between 1998 and 2007 and since then the techniques and materials used in the recanalisation of CTO have been substantially extended and improved. We therefore reevaluated the issue of the impact of gender on procedural characteristics, complications and acute results following CTO-PCI for a cohort of 780 patients between 2010 and 2015.

## Methods

A total of 780 consecutive patients were included in this study between 2010–2015. They underwent PCI for at least one CTO in one high-volume centre with two experienced operators. Indications for inclusion were angina pectoris and/or a positive functional ischaemia test by magnetic resonance imaging (MRI) or transthoracic echocardiography in the territory of the occluded artery of more than 10%. Both antegrade and retrograde CTO techniques were applied, and the procedures were performed in a standardised manner. Of the patients, 63% underwent a first-attempt CTO-PCI.

Without a prior angiogram it proved difficult to determine the duration of the CTO. Then estimation of the occlusion duration was based upon the first onset of angina or dyspnoea. In some cases there was also a history of acute myocardial infarction in the target vessel territory.

The mean Japanese Chronic Total Occlusion Score (J-CTO Score) was 2.8 and 2.9 respectively proving that gender-related differences in CTO complexity did not exist. To prevent thromboembolic complications, heparin was given during the interventions guided by the activated clotting time (>250 sec). All procedures were performed via both the femoral arteries using 7‑French guiding catheters; in the majority of cases bilateral injections of contrast fluid were given to determine the length of the lesion and the existence and extent of intercoronary collaterals. Only drug-eluting stents were implanted. After PCI a dual antiplatelet therapy consisting of 100 mg of aspirin once daily indefinitely and 75 mg clopidogrel daily for at least 6 months was continued. An Angio-Seal vascular closure device (St. Jude Medical, USA) was used after the arterial puncture.

Procedural success was defined as successful recanalisation of the CTO with residual stenosis <30% and restoration of TIMI flow grade 3. A composite safety endpoint summarising severe complications such as cardiovascular mortality, vessel perforation, cardiac tamponade, myocardial infarction and stroke was evaluated for each patient.

### Statistical analysis

Continuous data are presented as the mean ± standard deviation or as the median with the interquartile range; categorical data are presented as numbers and percentages unless otherwise specified. We used unpaired t tests for parametric variables, Mann-Whitney U tests for nonparametric variables, and Pearson’s chi-square tests for categorical variables to perform pairwise comparisons by sex and by PCI success for baseline and procedural characteristics. The comparisons of patient groups with and without PCI success were performed for all patients together and in subgroups by sex. An unadjusted odds ratio (OR, female vs. male) for success of the event PCI was estimated using a univariable logistic regression model: a risk-adjusted OR using a multivariable logistic regression model. Clinically relevant patient characteristics showing a difference in pairwise comparisons by sex (*p*-value <0.05) and remaining in the model after a backward, stepwise selection procedure were included in the final model. The variables considered are given in Table 1.

**Table 1:** Baseline and procedural characteristics by sex

	Female (*n* = 126)	Male (*n* = 654)	*p*-value
Age	66.9 ± 10.6	61.1 ± 10.4	<0.001^a^
Hypertension	81 (82.7)	427 (80.7)	0.654^c^
Diabetes	29 (29.6)	141 (26.7)	0.548^c^
Smoking	22 (22.4)	189 (35.7)	0.011^c^
COPD	7 (7.1)	42 (7.9)	0.787^c^
BMI	28.2 ± 4.6	28.3 ± 4.0	0.921^a^
PAD	15 (15.3)	57 (10.8)	0.194^c^
Family history	32 (32.7)	129 (24.4)	0.085^c^
Prior MI	44 (45.4)	278 (52.5)	0.199^c^
Ejection fraction	59.8 ± 9.5	57.2 ± 10.8	0.012^a^
Multivessel disease	93 (73.8)	500 (76.8)	0.469^c^
CTO location	–	–	0.067^c^
**–** LAD	43 (34.1)	190 (29.3)	–
– LCX	10 (7.9)	102 (15.7)	–
– RCA	73 (57.9)	356 (54.9)	–
Length of CTO	40 (30–50)	40 (30–50)	0.204^b^
Calcification	87 (69.0)	422 (64.5)	0.329^c^
Tortuosity	56 (44.4)	312 (47.7)	0.502^c^
Retrograde	26 (20.6)	131 (20.0)	0.877^c^
Stent type	–	–	0.207^c^
– DES	96 (94.1)	499 (96.7)	–
– BMS	6 (5.9)	17 (3.3)	–
Number of stents	2 (2–3)	2 (2–3)	0.705^b^
Diameter of stent	3 (2.5–3)	3 (3–3.5)	<0.001^b^
Length of stent	63.5 (40–84)	62 (44–84)	0.500^b^
Amount of contrast medium	250 (170–330)	250 (200–350)	0.218^b^
Fluoroscopy time	31.5 (21–43)	31 (21–46)	0.726^b^
Examination time	90 (60–120)	90 (70–120)	0.638^b^

Data presented as *n* (%), mean ± standard deviation or median (25th–75th percentile)

*BMI* body mass index, *BMS* bare metal stent, *COPD* chronic obstructive pulmonary disease, *DES* drug-eluting stent,* LAD* left anterior descending, *LCX* left circumflex, *MI* myocardial infarction, *PAD* peripheral arterial disease, *RCA* right coronary artery

^a^t test; ^b^Mann-Whitney U test; ^c^Pearson’s chi-square test

For logistic regression models, ORs, corresponding 95% confidence intervals and *p*-values (Wald test) are given. All reported *p*-values are two-sided and *p*-values <0.05 are regarded as statistically significant. Because the analyses were regarded as explorative, we did not adjust for multiple testing. Statistical analyses were performed using IBM-SPPS Statistics version 23 (Armonk, NY).

### Materials

A robust back-up of the guiding catheter is a necessary prerequisite for CTO-PCI. For the left coronary artery an EBU catheter, for the right coronary artery either a JR4, an IMA, a Multipurpose or an Amplatz catheter were applied. The selection of coronary guide wires followed a standardised concept of a ‘step-up’ guidewire strategy starting with tapered polymer soft tip and ending up with super-stiff guidewires (up to 12-g wires). For the selection of microcatheters it is important that they have a low outer diameter and allow wire manoeuvrability. Two kinds of microcatheters were used: the Finecross microcatheter (Terumo, Japan) for the antegrade approach, and the Corsair (Asahi Intecc, Japan) microcatheter for the retrograde access.

### Techniques

CTO-PCI was started mostly on the antegrade route. Bilateral contrast fluid injection allows to visualise the track in detail, identify potential collaterals and guide the process at any stage [7]. At the beginning a soft tipped tapered hydrophilic wire was used to identify microchannels for entering and crossing the occlusion. The crossing process was further supported by contrast fluid injections into the proximal cap through the distal microcatheter [8]. The wire stiffness was increased stepwise. If the CTO could not be crossed using the routine techniques, the parallel wire technique, the see-saw wire technique, the anchor wire or anchor balloon technique were applied [9, 10]. If required the manoeuvres were guided by intravascular ultrasound (IVUS) to understand the local anatomy and identify the exact entry point of the CTO. The retrograde approach was chosen if the antegrade approach failed [11].

The basis for the retrograde approach is septal and epicardial coronary collaterals or bypass grafts. There is a huge variety in the size, extent and anatomical course of the septal collaterals. They may have a straight and visible connection to the recipient vessel but in about 50% of the cases, they do not provide access to the distal CTO segment. Many collaterals are even angiographically invisible. For a successful use their diameter and tortuosity are most important. Techniques used for the retrograde approach were the standard ‘true’ retrograde wire crossing, the kissing wire technique, the controlled antegrade and retrograde tracking (CART), and the reverse CART techniques with or without a knuckle wire.

With the standard true retrograde technique, the retrograde wire is advanced through the collaterals and the lesion and threaded into the antegrade guide so that it could be trapped in the antegrade guide. If this approach fails, the kissing wire technique is the next choice; in this technique the retrograde wire is advanced to the proximal part and proximal cap within the lesion, then both the antegrade and the retrograde wire can meet within the CTO lesion.

A further technique is the reverse CART technique. In reverse CART a small balloon is inflated in the CTO over an antegrade guidewire to create a subintimal or intimal disruption and a connection with the retrograde guidewire, which then facilitates the retrograde guidewire to pass into the proximal true lumen [12]. Here IVUS guidance using an antegrade approach is often a helpful tool.

A ‘fully hybrid’ approach, which combines several approaches such as the antegrade and retrograde, a wire escalation strategy and a dissection-reentry, was not used in this study [13].

## Results

Of the 780 patients, 126 (16%) were female and 654 (84%) male. Baseline and procedural characteristics of both groups undergoing attempted CTO recanalisation are summarised in Table 1.

Male patients were younger than women (61.1 ± 10.4 years vs. 66.9 ± 10.6 years; *p* < 0.001). With regard to cardiovascular risk factors, men were more often smokers (35.7% vs. 22.4%; *p* = 0.011), but less often had a history of coronary artery disease (CAD) compared with female patients (24.4% vs. 32.7%; *p* = 0.085) who suffered more from diabetes mellitus (29.6% vs. 26.7%; *p* = 0.55) and atrial hypertension (82.7% vs. 80.7%; *p* = 0.55). The length of the occlusion was similar between the two groups (40 mm) but women frequently had severe calcification of the CTO (69.0 vs. 64.5%; *p* = 0.33).

Women with a successful CTO recanalisation had a comparable left ventricular ejection fraction (EF) (60.1 ± 9.6% vs. 58.3 ± 9.1%; *p* = 0.39) and family history of CAD (27% vs. 5%; *p* = 0.51) to female patients with a failed procedure. Compared with those with an unsuccessful intervention, men with a successful CTO-PCI less frequently had a prior myocardial infarction (49.1% vs. 65.7%; *p* = 0.002), a better ejection fraction (58.0 vs. 53.8%; *p* < 0.001) and more often had a family history of CAD (26.5% vs. 16.0%; *p* = 0.03).

There were no differences with respect to the amount of contrast medium (250 ml (170–330) vs. 250 (200–350); *p* = 0.22), fluoroscopy time (31.5 min (21–43) vs. 31.0 min (21–46); *p* = 0.73) and examination time (90 min (60–120) vs. 90 min (70–120); *p* = 0.638) as well as to the length of the stent (63.5 vs. 62 mm; *p* = 0.5) or the number of the stents (2 (2–3) vs. 2 (2–3); *p* = 0.705). The stent diameter was slightly smaller in women (2.5–3.0 mm vs. 3.0–3.5 mm; *p* < 0.001) which was not surprising because the lumen calibre tends to be smaller in women than in men (Table 2; [14]).

**Table 2:** Baseline and Procedural characteristics by PCI success and sex

	Successful PCI total (*n* = 626)	Failed PCI total (*n* = 154)	*p*-value	Successful PCI female (*n* = 102)	Failed PCI female (*n* = 24)	*p*-value	Successful PCI male (*n* = 524)	Failed PCI male (*n* = 130)	*p*-value
Age	62.0 ± 10.5	62.3 ± 11.4	0.755^a^	66.7 ± 10.6	67.8 ± 10.6	0.646^a^	61.1 ± 10.2	61.2 ± 11.2	0.869^a^
Hypertension	411 (81.9)	97 (77.6)	0.276^b^	68 (86.1)	13 (68.4)	0.068^b^	343 (81.1)	84 (79.2)	0.667^c^
Diabetes	137 (27.3)	33 (26.4)	0.841^b^	23 (29.1)	6 (31.6)	0.833^b^	114 (27.0)	27 (25.5)	0.758^c^
Smoking	168 (33.5)	43 (34.4)	0.843^b^	19 (24.1)	3 (15.8)	0.438^b^	149 (35.2)	40 (37.7)	0.629^c^
COPD	36 (7.2)	13 (10.4)	0.229^b^	7 (8.9)	0	0.178^b^	29 (6.9)	13 (12.3)	0.066^c^
BMI	28.2 ± 4.1	28.5 ± 4.4	0.368^a^	28.1 ± 4.3	29.0 ± 5.6	0.360^a^	28.2 ± 4.0	28.4 ± 4.2	0.582^a^
PAD	54 (10.8)	18 (14.3)	0.266^b^	12 (15.2)	3 (15.8)	0.948^b^	42 (9.9)	15 (14.0)	0.223^c^
Family history	139 (27.7)	22 (17.6)	0.021^b^	27 (34.2)	5 (26.3)	0.512^b^	112 (26.5)	17 (16.0)	0.025^c^
Prior MI	239 (47.7)	83 (65.9)	<0.001^b^	32 (40.5)	12 (66.7)	0.044^b^	207 (49.1)	71 (65.7)	0.002^c^
EF	58.3 ± 10.3	54.5 ± 11.4	<0.001^a^	60.1 ± 9.6	58.3 ± 9.1	0.390^a^	58.0 ± 10.4	53.8 ± 11.7	<0.001^a^
Prior CABG	25 (7.8)	3 (5.1)	0.462^b^	1 (1.9)	1 (12.5)	0.121^b^	24 (9.0)	2 (3.9)	0.228^c^
Multivessel	480 (76.8)	113 (74.3)	0.523^a^	74 (72.5)	19 (79.2)	0.507^a^	406 (77.6)	94 (73.4)	0.314^c^
CTO location	–	–	0.086^a^	–	–	0.021^a^	–	–	0.032^c^
– LAD	195 (31.4)	38 (24.8)	–	33 (32.4)	10 (41.7)	–	162 (31.2)	28 (21.7)	–
– LCX	94 (15.1)	18 (11.8)	–	9 (8.8)	1 (4.2)	–	85 (16.4)	17 (13.2)	–
– RCA	332 (53.5)	97 (63.4)	–	60 (58.8)	13 (54.2)	–	272 (52.4)	84 (65.1)	–
Length of CTO	40 (30–50)	50 (40–60)	<0.001^b^	40 (30–50)	45 (40–50)	0.070^b^	40 (30–50)	40 (20–50)	<0.001^b^
Calcification	375 (59.9)	134 (87.0)	<0.001^a^	66 (64.7)	21 (87.5)	0.030^a^	309 (59.0)	113 (86.9)	<0.001^c^
Tortuosity	277 (44.2)	91 (59.1)	0.001^a^	41 (40.2)	15 (62.5)	0.048^a^	236 (45.0)	76 (58.5)	0.006^c^
Retrograde	97 (15.5)	60 (39.0)	<0.001^a^	15 (14.7)	11 (45.8)	0.001^a^	82 (15.6)	49 (37.7)	<0.001^c^
Amount of contrast medium	250 (200–350)	273 (200–400)	0.180^b^	250 (170–350)	250 (175–315)	0.975^b^	250 (200–350)	285 (200–400)	0.147^b^
Fluoroscopy time	28 (19–42)	40.5 (31–54)	<0.001^b^	27.5 (18–43)	36 (29–50)	0.016^b^	28 (19–42)	41.5 (33–56)	<0.001^b^
Examination time	90 (60–120)	90 (70–130)	0.009^b^	90 (60–120)	85 (70–130)	0.765^b^	90 (60–120)	93 (70–130)	0.005^b^

Data presented as *n* (%), mean ± standard deviation or median (25^th^ –75^th^ percentile)

*BMI* body mass index, *EF* ejection fraction, *CABG* coronary artery bypass graft, *COPD* chronic obstructive pulmonary disease, *LAD* left anterior descending, *LCX* left circumflex, *MI* myocardial infarction, *PAD* peripheral arterial disease, *RCA* right coronary artery.

^a^t test; ^b^Mann-Whitney U test; ^c^Pearson’s chi-square test

**Fig. 1:**
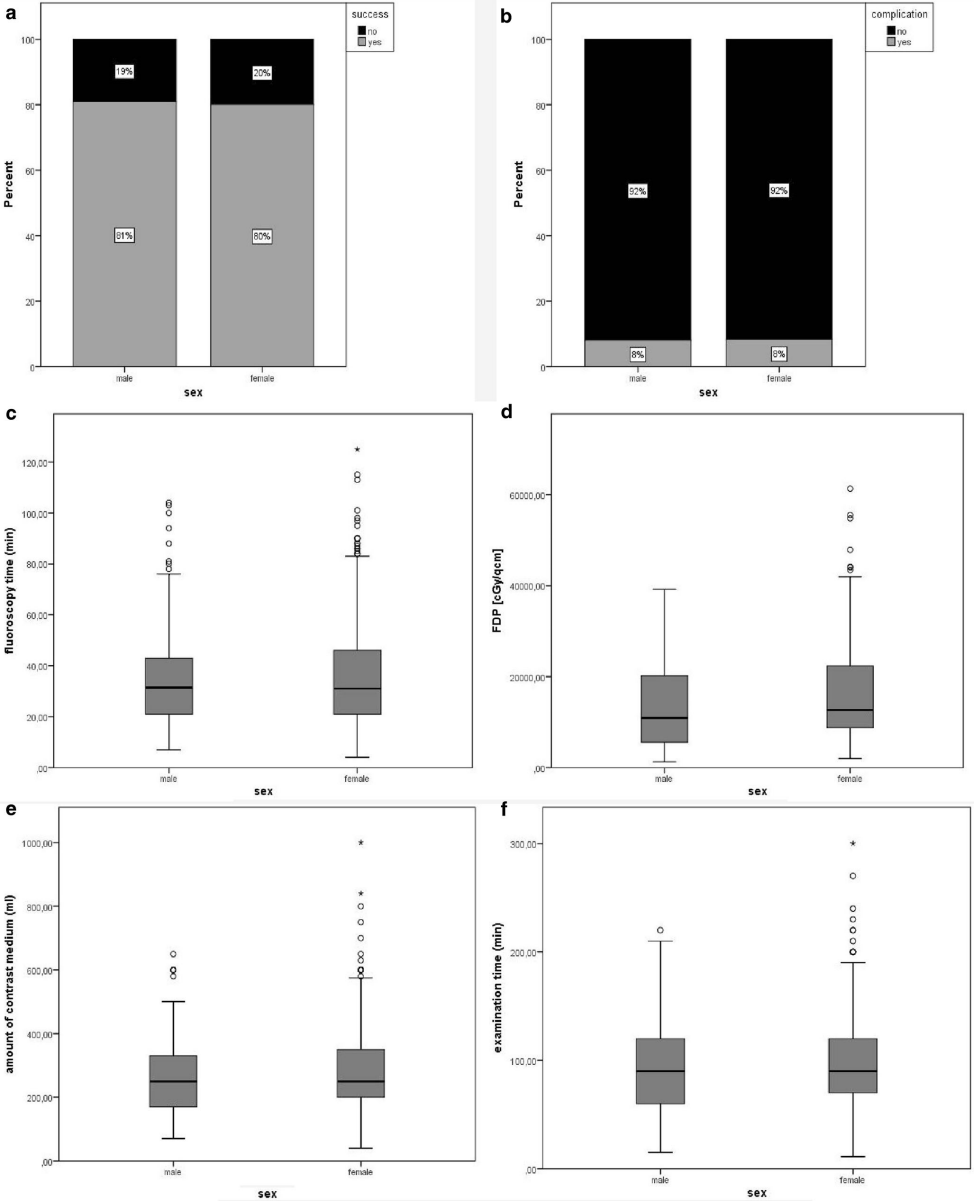
Comparison of female and male patients regarding **a** success, **b** complications, **c** fluoroscopy time, **d** dose area product (FDP), **e** amount of contrast medium, **f** examination time

The success rates were comparable in women and men. A total of 81.0% of the women and 80.1% of the male patients had a successful intervention, and the unadjusted OR was 1.054 (95% CI 0.650 to 1.711; *p* = 0.830; female vs. male). Age, smoking and ejection fraction were included into the multivariable analysis. After model selection, only ejection fraction remained in the model (OR 1.031, 95% CI 1.013 to 1.049, *p* = 0.001); and the revealed risk-adjusted OR for gender was 0.956 (95% CI 0.551 to 1.658, *p* = 0.872). There were no differences in the selection of materials used in women and men. In-hospital complication rates were very low in both groups (8.4% vs. 8.1%; *p* = 0.9) with no difference in statistical significance (Fig. 1). They included mainly vascular complications such as haematoma and cardiac tamponade which could be treated with a pericardiocentesis and without severe consequences (Table 3).

**Table 3:** In-hospital clinical events

	Female (*n* = 126)	Male (*n* = 654)
In-hospital death	0	0
Myocardial infarction	1	5
Stroke	1	5
Vascular complication	2	12
Cardiac tamponade	2	12
Coronary perforation	4	16
Emergent CABG	0	1

*CABG* coronary artery bypass graft

## Discussion

The impact of gender referring to outcome in cardiovascular diagnostic and therapeutic strategies has so far only been fragmentarily evaluated. We report gender-based acute outcome in a large consecutive series of patients undergoing recanalisation of at least one CTO. Only 16% of the patients were female but this is consistent with other trials suggesting women are less likely to undergo treatment of CAD. We did not find a significant difference in stent length, which supports the data of Claessen et al. and Miyauchi et al. [6, 24].

In previous PCI trials higher procedural mortality rates, more strokes and higher vascular complications were registered in women but newer data based on improved techniques and advanced materials revealed that these differences no longer exist [15–17]. Duvernoy et al. demonstrated that the relationship between female gender and increased risk of death and MACCE is no longer evident after elective PCI.

On the other hand the worse outcome of women after CABG surgery compared with men makes it necessary to provide alternatives if medically feasible [18–21]. Den Ruijter et al. showed that women were more likely to experience the composite endpoint including cardiovascular death, myocardial infarction and stroke.

Particularly the fact that women have a higher operative mortality then men after CABG surgery needs to bring up new strategies [22]. Blankstein et al. proved that operative mortality for the entire population was 2.88% (women 4.24% vs. men 2.23%; *p* < 0.0001) and 22% higher in women after a complete risk adjustment.

In contrast to these findings, we were able to show that a complex interventional procedure such as CTO-PCI can be performed safely in women with feasible acute results. CTO-PCI not only improves survival and left ventricular function but also gives freedom from angina pectoris and reduces the need for CABG [23]. Therefore, our data suggest that revascularisation of CTO should probably be considered more often as alternative treatment in women, whenever it can be performed to avoid surgical treatment and to achieve a complete revascularisation because we know that females benefit from complete revascularisation equally to males [24].

Women are on average older than men when they first undergo invasive cardiovascular procedures, presumably because of the protective effects of oestrogen against coronary atherosclerosis until menopause [25]. Consistent to previous studies the women in our study had a higher risk profile for cardiovascular diseases. Only a minority of patients in our study were female, which is comparable with the data of the current literature and might be a hint that the screening of patients with CAD between men and women is different.

Currently the lack of experienced operators and the low reimbursement are the main obstacles for a change in strategy.

### Study limitations

There are several limitations to our study. This is a retrospective study, and the absolute figures in the section ’successful PCI vs. failed PCI in female patients’ are low, all data were collected from a single centre. The results of this study could be influenced by selection criteria, operator experience, and varying techniques used by the operators. Furthermore, there is no follow-up beyond the in-hospital phase and some data concerning the cardiovascular risk, such as cholesterol, kidney function or a prior stroke are not available.

## Conclusions

Our study, which included 780 patients, suggests that women and men have a comparable success and complication rate after recanalisation of CTO. These results are contrary to gender-dependent comparisons of surgical revascularisation strategies demonstrating a worse early outcome with higher operative mortality in women compared with men.
